# Norovirus strain types found within the second infectious intestinal diseases (IID2) study an analysis of norovirus circulating in the community

**DOI:** 10.1186/s12879-019-3706-z

**Published:** 2019-01-25

**Authors:** John P. Harris, Miren Iturriza-Gomara, David J. Allen, Susan Kelly, Sarah J. O’Brien

**Affiliations:** 1 University of Liverpool, Instutue of Population Health Sciences, Liverpool, UK; 2 University of Liverpool Institute of Global Health, Liverpool, UK; 3 London School of Hygiene and Tropical Medicine, Liverpool, UK; 4 NIHR HPRU in Gastrointestinal Infections, Liverpool, UK; 5 Modelling, Evidence and Policy Research Group, School of Natural and Environmental Sciences, Newcastle University, Newcastle upon Tyne, UK

## Abstract

**Background:**

Norovirus is the commonest cause of infectious intestinal disease (IID) worldwide. In the UK community incidence of norovirus has been estimated at 59/1000 population, equating to four million cases a year. Whilst norovirus infects people of all ages, a substantial burden occurs in infants and young children. The population of viruses found in sporadic cases among infants has been observed to be more diverse than that associated with outbreaks. In this study, we analysed norovirus-positive specimens collected during the second study of infectious intestinal diseases (IID2 Study) a national community cohort study conducted between April 2008 and August 2009 We examined the data for differences in circulating norovirus strains between two arms of a community cohort, and differences between genotypes and disease outcomes such as illness duration and symptom profiles.

**Methods:**

Analysis was conducted to assess genetic diversity of noroviruses in the community. We also assessed differences in the cycle threshold (Ct) value, as a proxy for viral load, between norovirus genogroups and genotypes, and differences in reported symptoms or length of illness in relation to genogroup and genotype.

**Results:**

There were 477 samples where norovirus was detected. Whilst 85% of people recovered within two days for vomiting; diarrhoea symptoms were reported to day 4 for 83% of the cases, and 10% of people reported symptoms of diarrhoea lasting between five and six days. Both diarrhoea and vomiting symptoms lasted longer in children aged < 5 years compared to adults. There was a significantly higher proportion of GII.4 in samples obtained from the GP arm of the study (chi-square = 17.8, *p* < 0.001) compared to samples received via post in the self-reporting arm. In the latter group, the prevalence of GII.6 was significantly higher (chi-square = 7.5, *p* < 0.001).

**Conclusions:**

We found that there is a difference in disease severity by age group. Children aged < 5 years had longer duration of illness, with 10% still having diarrhoea at seven days, and vomiting of between four and five days. The duration of illness reported is higher overall than one might expect for cases in the community in otherwise healthy individuals which has implications for infection control. No differences were observed in relation to duration of vomiting and or diarrhoea by genotype.

## Introduction

Norovirus is the commonest cause of infectious intestinal disease (IID) worldwide [1]. Viruses of the genus *Norovirus* (family *Caliciviridae*) have positive sense, single-stranded RNA genomes that exhibit high rates of mutation due to the error-prone nature of genome replication mediated by the low-fidelity viral RNA-dependent RNA polymerase. In turn, this generates a substantial amount of genetic diversity among the *Norovirus* genus: human norovirus strains predominantly belong to genogroup I (GI) and genogroup II (GII) which are subdivided into nine (GI.1-GI.9) and 22 (GII.1-GII.22) genotypes, respectively [2, 3]. Despite this high degree of diversity, norovirus strains of the GII.4 genotype are the most frequently detected worldwide [4–7]. The mechanisms by which GII.4 viruses persist and dominate in the population are not fully understood, but it is at least in part linked to the ability of these strains to rapidly mutate generating new antigenic profiles that are able to escape from population immunity [8–10].

In the UK, the Second Study of Infectious Intestinal Disease (IID2 Study), described in detail elsewhere [11, 12] utilised a prospective cohort design randomly selecting healthy people of all ages from randomly selected general practices (GP) across the UK with follow-up of volunteers at weekly intervals for one year to detect symptoms and determine the aetiology of cases of IID in the cohort, additionally cases who attended GP surgeries from volunteer practices also provided faecal specimens for analysis. A recent reanalysis of the IID2 Study data estimated the community incidence of norovirus to be 59/1000 population, equating to almost four million cases a year [13] .

From community-based studies such as the IID2 Study, and others [11, 14], it is increasingly clear that whilst norovirus infects people of all ages across the world, a substantial burden of norovirus-associated disease occurs in infants and young children [13, 15, 16]. It has been observed that the population of viruses found in sporadic cases among infants was more diverse than that associated with outbreaks [17]. These data indicate a need for better understanding of the molecular epidemiology of norovirus strains associated with sporadic cases (as well as outbreaks), and circulating among children and in the wider community.

Previous studies have shown an association between norovirus infection and poorer outcomes in hospitalised cases [18] and with increased age [19]. In this study, we analysed norovirus-positive specimens collected during the IID2 Study from either the GP presentation arm of the study or the Prospective Cohort arm to determine the diversity of norovirus genotypes associated with cases of norovirus-associated IID in the community. Further, we examined the data for differences in circulating norovirus strains between the two arms of the community cohort, and differences between genotypes and disease outcomes such as illness duration or symptom profiles (vomiting/diarrhoea etc).

## Methods

### Setting

Cases were drawn from the community, i.e. did not attend hospital, from two concurrent studies within the IID2 study, firstly, where participants visited their GP for symptoms related to infectious intestinal disease and secondly, those who were part of a volunteer cohort, recruited via GP surgeries and followed up at weekly intervals, who self-reported illness [12]. The latter cases were also asked about their contact with health services. For this study the second group are categorised as other community cases to distinguish them from those cases ascertained from the GP presentation arm of the IID2 study [12].

### Case definition

Cases were defined as people developing clinically significant vomiting (more than once in a 24 h period, or where it caused incapacity or was accompanied by other symptoms) or loose stools for a period of less than two weeks, without a known non-infectious cause and who had previously been symptom free in the preceding three weeks. Cases were asked to complete a clinical symptom questionnaire. If vomiting was associated with non-infectious causes such as pyloric stenosis or morning sickness, these were excluded from the case definition.

### Laboratory methods

Cases were asked to provide stool samples for microbiological examination. Diagnosis of norovirus was by real time quantitative reverse transcription polymerase chain reaction (RT-PCR) [12] . In the original study (IID2), clinically relevant cases of norovirus were defined as those where detection occurred with a cycle threshold (Ct) of < 30. This study attempted to genotype all of the stool samples in which norovirus was detected with a Ct value of < 40 as this value is more in line with that normally used in clinical diagnostic laboratories [13] . Genotyping was performed by amplification of the S domain encoding region of the VP1 gene (ORF2, region C) [20], followed by direct Sanger sequencing, and genotypes were assigned as described elsewhere [21].

### Statistical analysis

Initial analysis was conducted to assess which genotypes occurred in the community and if these differed between those attending their GP and those who self-report illness. We also assessed differences in Ct values as a proxy for viral load, between the genogroups and genotypes. Analysis was conducted where there were at least five samples with a measureable Ct value in each genotype. Data were also investigated to assess differences in self-reported symptoms, gathered by standard questionnaire, or length of illness in relation to genogroup and genotype (chi square test). Analysis was conducted using the R statistical package [22].

## Results

There was a total of 477 samples where norovirus was detected. The distribution of samples by age at submission in cases is shown in Table 1. The greatest number of samples were in the youngest age group (0–4 years) and in adults aged 25–64 years. The fewest number of samples were from young adults aged 16–24 years (Table 1).

**Table 1:** Age distribution of participant’s samples

age group	Number of samples	Percent of total samples
0–4	121	25.37
5–15	50	10.48
16–24	10	2.10
25–49	100	20.96
50–64	116	24.32
65+	76	15.93
Not known	4	0.84
Total	477	100

**Fig. 1:**
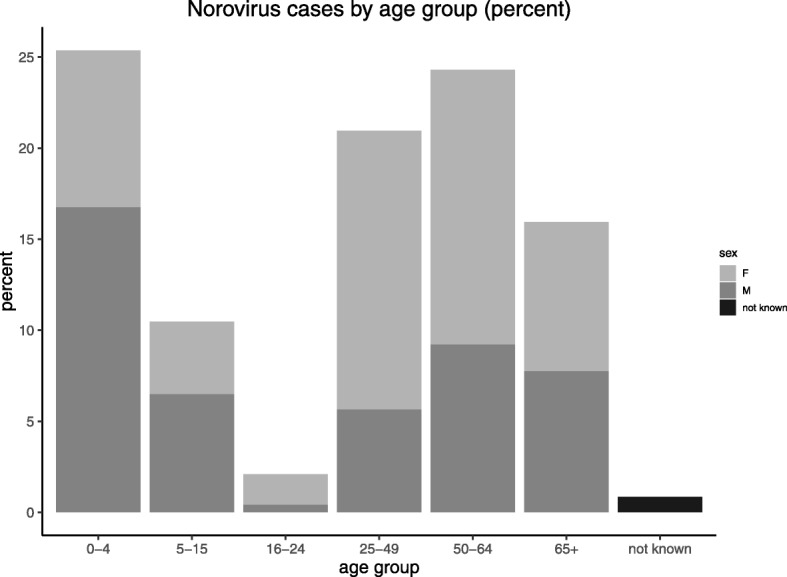
Norovirus cases by age group and sex

There were slightly more female participants overall with 52.83% females and 46.33% males. However, there were significant differences in the proportion of males and females by age group (chi square = 45, *p* < 0.001), with twice as many males compared to females in the youngest age group (0–4 years), In the 16–64 years age group there was a greater proportion of females (74%) than males. The older age group (> = 65 years) males and females were equally represented (Fig. 1).

Table 2 shows the distribution of genotypes by age and surveillance arm. The majority of samples were GII (91.4%). There were 40 (8.39%) GI and one sample was mixed GI and GII. Ninety four percent (448) (93.9%) were assigned to a single genotype, and 28 samples could not be typed (6.1%). In those samples identified as GII, the majority were GII.4 (52.29%) whereas for GI samples GI.3 and GI.4 were the predominant types (32.5 and 22.5% respectively).

**Table 2:** Distribution of genotypes by age group and surveillance arm

	Genotype																	
Surveillance arm/age group	GI.2	GI.3	GI.4	GI.5	GI.6	GI Untyped	GII.1	GII.13	GII.2	GII.3	GII.4	GII.6	GII.7	GII.8	GII.9	GII Untyped	Mixed genotype^a^	Total
GP arm																		
0–4	–	–	–	–	–	1	2	–	–	13	27	5	1	–	–	3	–	52
05–15	–	1	1	–	–	–	–	–	–	–	2	4	1	–	–	–	–	9
16–49	–	2	-	–	1	–	1	–	2	2	21	4	2	1	–	2	–	38
50–64	–	1	1	–	1	1	3	–	–	2	26	–	–	–	–	–	–	35
65+	–	-	1	–	–	–	–	–	–	1	24	1	–	1	–	–	–	28
Not known	–	-	-	–	–	2	–	–	–	–	–	–	–	–	–	–	–	2
Sub total	–	4	3	–	2	4	6	–	2	18	100	14	4	2	–	5	–	164
Self-reporting arm																		
0–4	–	–	2	–	–	1	3	–	7	7	26	14	5	–	–	3	1	69
05–15	1	2	–	1	–	1	6	–	7	1	8	10	2	1	–	1	–	41
16–49	–	3	–	–	1	2	4	1	5	7	31	13	1	–	–	4	–	72
50–64	1	2	1	–	–	2	8	–	3	9	38	9	4	1	1	2	–	81
65+	–	2	2	–	–	2	4	–	2	1	24	10	–	–	–	1	–	48
Not known	–	–	1	–	–	–	–	–	–	–	1	–	–	–	–	–	–	2
Sub total	2	9	6	1	1	8	25	1	24	25	128	56	12	2	1	11	1	313
Total	2	13	9	1	3	12	31	1	26	43	228	70	16	4	1	16	1	477

^a^Note: the mixed genotype sample was: GI-3 / GII-6

There was a significantly higher proportion of GII.4 in samples obtained from the GP arm of the study (chi-square = 17.8, *p* < 0.001) compared to samples received via post in the self-reporting arm. In the latter group, the prevalence of GII.6 was significantly higher (chi-square = 7.5, *p* < 0.001).

**Fig. 2:**
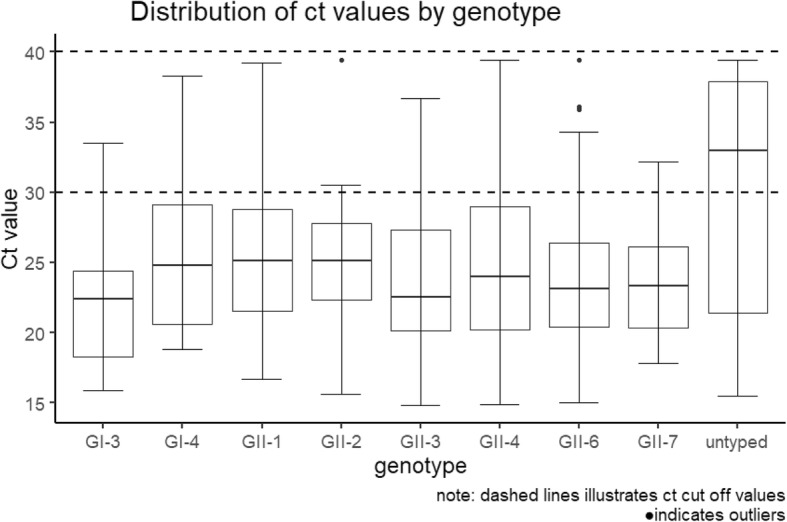
Distribution of ct values by genotype

**Fig. 3:**
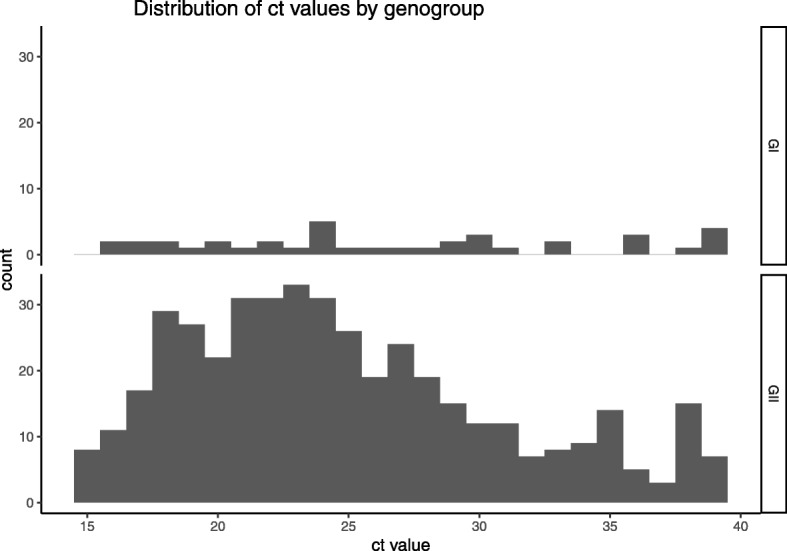
Distribution of ct values by genogroup

There was no statistically significant difference in the Ct values between the genotypes, however the median Ct value for those samples from which a genotype could not be determined was higher than those samples that were genotyped. The Ct values for specimens where the virus could not be genotyped clustered towards higher values (Inter Quartile Range (IQR) 21.42–37.9), and the median value 32.95 was above the cut off value of 30 [23] used in the IID2 study as a measure of symptomatic infection (Figs. 2 and 3).

Analysis of symptoms showed that vomiting and diarrhoea were the commonest symptoms reported (Table 3). Other commonly reported symptoms were nausea, loss of appetite and abdominal pain. There was no difference in the proportion of reported symptoms by virus genogroup or genotype (Chi square = 11.75, *p* = 0.761, note chi square test excludes the mixed category). Diarrhoea symptoms lasted for an average of 2.8 days (median 2, IQR 1–4) and vomiting symptoms lasted for an average of 1.7 (median 1 IQR 1–2).

**Table 3:** Symptoms by genogroup/genotype

Genogroup	Diarrhoea	Vomiting	Diarrhoea & vomiting	Abdominal Pain	Nausea	Loss of appetite	Fever	Headache	Sinus^a^	Total
GI	11	5	18	19	26	29	18	15	12	40
GII4	55	22	128	128	146	178	65	83	57	228
GIInot4	38	30	98	91	110	137	60	61	58	208
mixed	0	0	1	0	0	1	1	1	1	1

^a^Cough/runny or blocked nose or sore throat

**Fig. 4:**
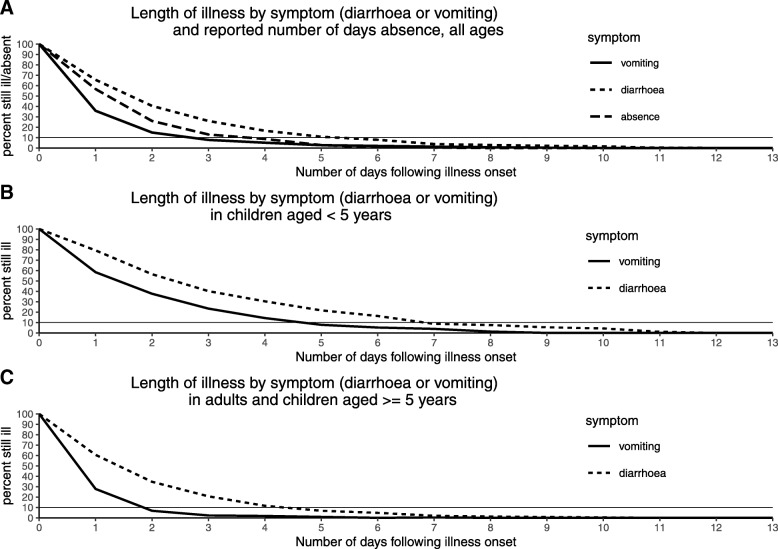
Length of time for recovery from symptoms of norovirus **a** All ages **b** aged < 5 years **c** ages > = 5 years

Whilst 85% of people reported recovery within two days for vomiting, diarrhoea symptoms were reported to day 4 for 83% of the cases. Ten percent of people had symptoms of vomiting lasting 2 to 3 days, whereas 10% of people reported symptoms of diarrhoea lasting between five and six days. The median reported length of absence from work or school was 2 days with 87% of people reporting having three or fewer days absence (Fig. 4). Both diarrhoea and vomiting symptoms lasted longer in children under 5 than in adults.

## Discussion

In this study we have shown that community sporadic cases of norovirus infections are dominated by genogroup II noroviruses (88%), this is in line with other studies [4, 5, 24] and also with the data from national surveillance in England [25]. National surveillance primarily represents norovirus outbreaks, and predominantly from disease associated with health care settings. Data from national surveillance during the same period in which the IID-2 sample collection (data not shown) took place showed that GII strains represented 91% of the total of strains received. In this study 6% of the strains were GI (compared to 9% in the national surveillance) and among both data sets GI-3 was the predominant genotype (46% in the IID study and 44% for national surveillance). Genogroup II viruses were dominated by genotype II.4 (54% in the IID cohort and 84% in national surveillance) There was a significant difference in the proportion of genogroup II.4 found in those where samples were taken from patients attending their GP compared to other community sources.

In this study formal assessment of disease severity was not carried out, and although no differences were observed in relation to duration of vomiting and or diarrhoea by genotype, we found that there is a difference in disease severity by age group. Whilst, most people had recovered from their symptoms within three days, a proportion of cases reported symptoms lasting longer than this. For example, 10% of patients reported diarrhoea symptoms at between five and six days duration. Children aged < 5 years had longer duration of illness, with 10% still having diarrhoea at seven days, and vomiting of between four and five days. In older children and adults these figures were four and two days respectively. The proportion of people reporting diarrhoea symptoms lasting four days or more is a concern for control of infectious diseases especially for adults involved in preparation of food or those who are involved in health care roles. Nevertheless, the duration of illness reported is higher than one might expect for cases in the community given that these people are generally healthy. Hospitalised patients are known to have a longer duration of illness compared to health care workers and care home patients and this is likely related to their underlying health [18]. Our findings and also the fact that most hospital or healthcare associated outbreaks of norovirus (from national surveillance) tend to be predominantly associated with GII.4 norovirus, suggest that GII.4 might be associated with more severe disease requiring medical attention. This might also reflect issues around understanding when diarrhoea stops and simply how long the bowel takes to settle down after infection. It is easy to state when vomiting has ceased, perhaps less clear when diarrhoea ends.

There was no statistical difference in the Ct values by genotype. Genotypes GI.3 and GII.3 had the lowest median Ct values 22.4 and 22.5 respectively. Samples that were un-typed had the highest median Ct value at 33. There were only 26 samples in this group, and 50 % of these were between 33 and 39.4, and only four samples had Ct values below 20. The high median value of this group suggests the samples had a low viral load and this might explain why they were not able to be genotyped. Young children (aged < 5 years) also had lower median Ct values than other age groups but there was no relationship between age and genotype. Young children (aged < 5 years) were the largest single age group in the analysis followed by older adults (aged 50–64 years). This is not surprising given that norovirus rates are higher in those aged < 5 years [13]. One reason for higher rates of infection in children could be associated with severity of symptoms, for example; if symptoms persist for more than two or three days, parents might be more likely to contact medical services. An interesting point is that the proportion of males in the youngest age groups was greater than females, in the age group 0–4 years the ratio of males to females was 2:1 and in the 5–15 years age group the ratio was 1.6:1. In older age groups this ratio changed and females were more greatly represented than males. In the 16 to 49 age group the ratio was almost 3:1 in favour of females. The oldest age group saw equal proportions of males and females. The drivers of these differences in ratios for males to females in the different age groups is difficult to explain from this data. Animal model data suggests sex differences for infections in animals implicating the role of male sex hormones in increasing susceptibility to infection in males. There are differences in the developing immune systems of the sexes in humans related to differences in sex hormones which can affect immunity in infants and the very young and therefore, differential observations of infectious diseases between the sexes [26]. It should be noted that data from Public Health England (PHE) on norovirus laboratory reports suggests that the male/female ratio is nearer parity in young children aged < 10, and slightly increased numbers of females in age groups of young adults to age 59 and a much greater proportion of females in those aged 80 and over. Therefore one of the main limitations of the study is the lack of participation of young adults, particularly those aged 16–24 years and the differences in the sexes in the different age groups. This might be linked to characteristics of health seeking behaviour. It is also well documented that people with serious underlying health conditions shed norovirus in their stools for a long time [27, 28] and for the elderly or those with serious underlying conditions there is a measurable attribution to mortality [19]. This finding is likely a reflection on the proportion of very young.

A further limitation is that the typing of norovirus was conducted using only region C and as such although we describe capsid types, we are not able to report on polymerase types and recombinant strains circulating among the population surveyed. However, analysis of capsid types in this study aligns with methods used in national surveillance typing of norovirus in England and Wales, and so allows us to compare viruses circulating in the community with those associated with outbreaks, which are the majority of those collected through national surveillance. In future studies, this limitation is likely to be ameliorated as whole genome sequencing techniques for analysis of norovirus become more accessible, which will yield data on capsid, polymerase and recombinant genomes.

Despite the limitations, this study shows the prevalence of GII.4 noroviruses in the community from two community settings, GP and non-GP settings. Samples were taken within three days in non GP settings and within nine days of GP consultation. Furthermore illness can last for several days, longer than expected in otherwise healthy individuals which has implications for infection control.

## Abbreviations

Ct: Cycle threshold
GP: General practice/practitioner
HPRU: Health Protection Research Unit
IID: Infectious Intestinal Disease
NIHR: National Institute for Health Research
RT-PCR: Reverse transcription Polymerase Chain Reaction
